# Effects of the mandibular advancement device on daytime sleepiness, quality of life and polysomnographic proﬁle of public transport drivers with obstructive sleep apnea syndrome

**DOI:** 10.5935/1984-0063.20200058

**Published:** 2021

**Authors:** Antonio Luiz Rocha, Litiele Evelin Wagner, Dulciane Nunes Paiva

**Affiliations:** 1 Universidade de Santa Cruz do Sul (UNISC) - Programa de Pós-Graduação Mestrado e Doutorado em Promoção da Saúde (PPGPS). Santa Cruz do Sul, Rio Grande do Sul - Brazil.; 2 Universidade de Santa Cruz do Sul (UNISC) - Programa de Residência Multiprofissional em Saúde do Hospital Santa Cruz (HSC). Santa Cruz do Sul, Rio Grande do Sul - Brazil.; 3 Universidade de Santa Cruz do Sul (UNISC) - Departamento de Ciências da Saúde e Programa de Pós-Graduação Mestrado e Doutorado em Promoção da Saúde (PPGPS). Santa Cruz do Sul - RS - Rio Grande do Sul - Brazil.

**Keywords:** Sleep Apnea Syndromes, Sleepiness, Quality of Life

## Abstract

**Objective:**

To evaluate the effects of the mandibular advancement device (MAD) on daytime sleepiness, quality of life (QoL) and polysomnographic profile of intercity transport drivers with obstructive sleep apnea syndrome (OSAS).

**Material and Methods:**

A quasi-experimental study evaluating intercity transport drivers from March to September 2019. The apnea-hypopnea index (AHI) was evaluated by type III polysomnography, which defined the severity of the disease. OSAS: mild (5 to 15), moderate (15 to 29), or severe (= 30). Sleepiness was assessed using the Epworth sleepiness scale, consisting of 8 questions about the likelihood of drowsiness in daily situations. QoL was assessed using the SF-36 questionnaire, which provides the score in eight domains: functional capacity, physical aspects, pain, general health status, vitality, social aspects, emotional aspects, and mental health. Drivers with OSAS underwent intervention with application of personalized MAD for 8 to 12 weeks.

**Results:**

The total sample (n=23) (44.77±11.56 years) had a body mass index (BMI) of 30.64±4.66kg/m2, and an OSAS prevalence of 65.2% of drivers (n=15). There were losses of 4 drivers so that the ﬁnal sample of drivers with OSAS for the intervention with the MAD was 11 individuals, with an average age of 45.54±9.41 years and BMI of 32.21±3.17kg/m². There was a decrease in AHI (28.51±15.66ev/h 012.11±6.70ev/h, p=0.002) and pain (60 (50-60)040 (40-50), p=0.015) after the intervention.

**Conclusion:**

There was a reduction in AHI in intercity transport drivers after implementing the MAD procedure.

## INTRODUCTION

Obstructive sleep apnea syndrome (OSAS) is characterized by an alteration in breathing during sleep in which there are repeated episodes of airway obstruction or narrowing resulting in apneas or hypopnea. This change can be attributed to anatomical changes in the upper airways and craniofacial skeleton, imbalances in the soft tissues and bone structures which surround the upper airways and cause a reduction in the size of the pharynx, in addition to factors such as obesity, male gender, craniofacial abnormality, nasal obstruction, endocrine abnormalities and family history^1^^,^^2^.

Apnea or hypopnea episodes can influence inflammatory, cardiovascular, neurocognitive and metabolic responses, which result in increased morbidity and mortality^3^. The symptoms commonly presented by an individual with OSAS are tiredness upon waking up and the sensation of non-restorative sleep (regardless of the duration of sleep), excessive daytime sleepiness and worsening quality of life (QoL). It is noteworthy that drowsiness and sleeping less than 7 hours/night are predisposed to a high risk of traffic accidents, as there is a reduction in alertness while driving, increasing the chances of the driver sleeping at the wheel^4^^,^^5^.

One way of assessing the probability of sleepiness in these individuals is through the Epworth sleepiness scale (ESS), which is conFigured as an instrument that is easy to use and reliable in this population^6^^,^^7^. Evaluating QoL is relevant due to the drowsiness and tiredness presented by drivers with OSAS in order to establish measures which promote improving this condition^8^. An assessment of the polysomnographic profile can be used by the apnea-hypopnea index (AHI), which provides information on the number of apnea events that occurs every hour^9^.

Alternatives that reduce the risk of OSAS and improve QoL should be instituted such as the mandibular advancement device (MAD), as public transport drivers have greater difficulty in regulating the protocol use of continuous positive airway pressure (CPAP) due to work shifts and their lifestyle. MAD acts by maintaining the mandible and tongue in the protruding position, culminating in an enlarged airway which reduces its collapse and is indicated for patients with mild and moderate OSAS^10^^,^^11^. In view of the above, this study aimed to evaluate the effects of MAD on the polysomnographic profile, daytime sleepiness and QoL of public transport drivers with sleep obstructive apnea syndrome.

## MATERIAL AND METHODS

This study enrolled intercity public transport drivers (males) from companies located in a city in the interior of Rio Grande do Sul, RS (n= 23), aged between 20 and 70 years old and diagnosed with OSAS by an experienced dental surgeon through polysomnographic examination.

The study was carried out between March and September 2019 and the individuals were selected by voluntary participation provided they met the inclusion criteria. Male individuals with a diagnosis of mild, moderate and severe OSAS were included. The presence of periodontal disease, little bone insertion of the teeth, with temporomandibular joint dysfunction, non-retentive teeth, small jaw propulsion capacity <6mm or those who did not sign the free and informed consent form (ICF) were excluded.

### Study design

This was a quasi-experimental study conducted in a dental clinic and at the research volunteer’s residence, with data analysis performed at the University of Santa Cruz do Sul, Brazil. An anthropometric evaluation was carried out with application of the ESS and the SF-36 quality of life questionnaire, as well as a type III polysomnographic examination.

All researchers were appropriately trained on how to perform the tests, use the instruments in accordance with quality criteria and were blinded regarding which group the patient belonged to for all the performed tests. The study was approved by the Research Ethics Committee of the University of Santa Cruz do Sul under protocol No. 3,078,259, and all participants signed a written informed consent form.

**Figure 1:**
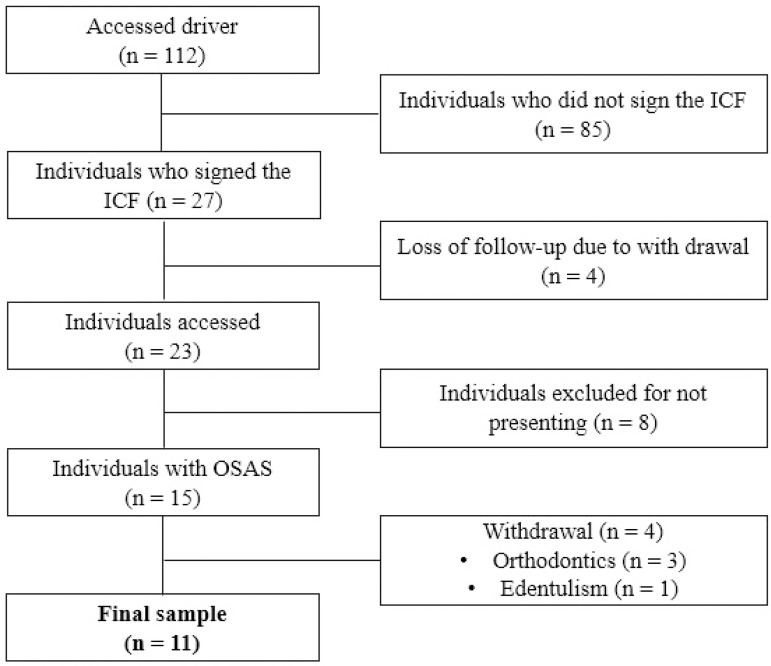
Flowchart representative of sample loss and individuals who participated in the study.

The initial sample consisted of 112 individuals, however only 11 were submitted to the intervention procedure after adopting the study inclusion criteria and due to the occurrence of losses, as presented in the study flowchart (Figure 1).

Body mass and height were assessed using an anthropometric mechanical scale (Filizola^®^, Brazil). The body mass index (BMI) was obtained using the classification recommended by the World Health Organization. In addition, waist circumference (WC) was measured at the midpoint between the tenth rib and the iliac crest, and the hip circumference (QC) was measured at the largest hip circumference. The waist-hip ratio (WHR) was then calculated by the ratio of the waist circumference in centimeters to the hip circumference in centimeters. Neck circumference was measured at the midpoint of the spine cervical to the anterior middle of the neck. All measurements were taken using an anthropometric tape (Sanny Medical^®^ model SN-4010, Brazil) with the individual in an orthostatic position.

### Assessment of sleepiness level

The sleepiness assessment was performed using the EES, which was applied before placing the mandibular advancement device and after removing it. This scale consists of 8 questions which demonstrate the probability of an individual’s sleepiness on a 0 to 3-point scale in situations such as: sitting and reading, watching television, sitting in a public place, walking in a car for an hour without stopping (passenger), sitting after lunch without drinking alcohol, or sitting in a car stopped in traffic for a few minutes. The score ranges from 0 to 24 points, and increased daytime sleepiness is indicated when ≥10^7^^,^^12^.

### Quality of life

The QoL assessment was performed using the SF-36 quality of life questionnaire before and after placing the mandibular advancement device. This instrument consists of 36 surveys, which assess QoL in eight domains: functional capacity, physical aspects, pain, general health, vitality, social aspects, emotional aspects, and mental health. A raw scale was used to calculate the final score, which can vary from 0 to 100 points in each domain, with 0 being the worst and 100 being the best in a given domain^13^^,^^14^.

### Global perception of change

Patients’ global impression of change (PGIC) is used to assess the perception of improvement of individuals undergoing some intervention in which they rate their improvement on a 7-item scale: 1 = no changes; 2 = almost the same, without any visible change; 3 = slightly better, but without significant changes; 4 = with some improvements, but the change did not represent any real difference; 5 = moderately better, with a slight but significant change; 6 = better, and with improvements which made a real and useful difference; 7 = much better, and with a considerable improvement that made all the difference^15^.

### Apnea and hypopnea index

The AHI was assessed by a type III polysomnographic exam (ApneaLink Air; ResMed, Australia), which consists of a home exam performed using a device that the individuals use while sleeping at night in their own home. The individual initially watched a video that instructed him to use the equipment, which was previously trained for its effective use. The severity of OSAS was defined by the AHI score as: mild = 5-15; moderate = 15-29; or severe = AHI≥30^9^^,^^16^. Furthermore, we evaluate the median and nadir of peripheral oxygen saturation (SpO_2_) and the oxygen desaturation index (ODI).

### Intervention

**Figure 2:**
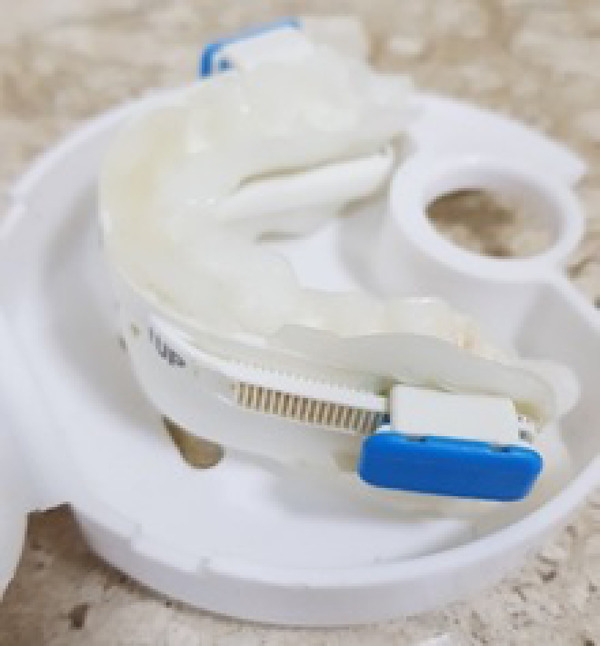
BluePro^®^ mandibular advancement device.

The MAD used in the present study was composed of thermoplastic material, treaTable and pre-fabricated, having sufficient retention forces to resist the opening forces of the mouth (Figure 2). Each patient used the referred mandibular advancement device (BluePro^®^; BlueSom, France) positioned by the dentist after performing the polysomnographic exam and verified OSAS. After adapting this boil and bite device, the individuals remained with it for 8 to no more than 12 weeks, with adjustments being made every 15 days according to the manufacturer’s instructions in order to promote maximum comfort to ensure the use for 8 to 12 weeks until the final polysomnographic exam, using the AHI success criteria of less than 5 events per hour^16^^-^^18^.

### Statistical analysis

The data were analyzed using the Software Statistical Package for Social Science (version 23.0, USA). The Shapiro-Wilk test was used to verify the normality of the distribution, with data presented as frequency, mean and standard deviation or median and interquartile range. The paired Student’s t-test was used for the parametric variables and the Wilcoxon signed-rank test for the non-parametric variables to compare the polysomnographic profile, daytime sleepiness and QoL before and after placing the mandibular advancement device. The Spearman’s correlation test (*p*<0.05) was used to correlate the variables.

## RESULTS

The characteristics of the sample are described in Table 1. The sample was 44.77±11.56 years old and had a BMI of 30.64±4.66kg/m^2^. It is noteworthy that there was a prevalence of overweight in 86.36% of the evaluated drivers (n=19) and an OSAS prevalence of 65.2% (n=15). The characteristics of drivers with OSAS undergoing the intervention procedure can be seen in Table 1. The average age was 45.54±9.41 years and the BMI was 32.21±3.17kg/m². There was a significant reduction in the AHI from the pre- to the post-intervention condition (*p*=0.002) and in the pain domain (*p*=0.015) of the SF-36 quality of life questionnaire, with no statistical difference in the other domains on the ESS and anthropometric measurements after the intervention (Table 2). However, it is noteworthy that 1 individual expressed feeling slightly better, 2 with some improvements, 3 moderately better, 4 felt improvement and 1 much better when the global perception of change was assessed by the PGIC.

**Table 1:** General sample characteristics.

Variables	(n=11)
Age (years)	45.54±9.41
Body mass (kg)	97.61±15.95
Height (m)	1.73±0.08
BMI (kg/m²)	32.21±3.17
Overweight	4
Obesity	7
**Anthropometric variables**	
NC (cm)	42.00 (41.00-43.50)
WC (cm)	110.13±9.27
HP (cm)	111.52±10.60
WHR (cm)	0.99±0.04
**Comorbidities, n**	
SAH	4
Cancer	1
Asthma	1
Profession time (years)	19.27±11.28
**Smoking, n**	
Yes	2
No	9
Smoking load (packs/year)	19.50±20.50

BMI: Body mass index; NC: Neck circumference; WC: Waist circumference; HC: Hip circumference; WHR: Waist-to-hip ratio; SAH: Systemic arterial hypertension; Data expressed as frequency, mean and standard deviation and median and interquartile range.

**Table 2:** Polysomnographic profile, daytime sleepiness and quality of life before and after the mandibular advancement procedure.

Variables	Pre	Pos	*p*-valor
AHI (ev/h)	28.51±15.66	12.11±6.70	0.002^a^
SpO2 nadir (%)	80.54±5,20	83,09±3,50	0,056a
SpO2 median (%)	92.72±1.42	93.27±0.90	0.111a
ODI (ev/h)	30.60±15.81	15.17±5.35	0.007a
Epworth	8.90±5.30	7.40±4.63	0.108^a^
Quality of life SF-36			
Capacity functional	80.00 (70.00-85.00)	80.00 (75.00-95.00)	0.365b
Physical limitations	75.00 (50.00-100.00)	100.00 (75.00-100.00)	0.200b
Pain	60.00 (50.00-60.00)	40.00 (40.00-50.00)	0.015b
General state	48.54±10.78	54.54±11.05	0.108^a^
Vitality	55.00±8.36	58.18±7.50	0.319^a^
Emotional aspects	100.00 (62.50-100.00)	100.00 (66.66-100.00)	0.273b
Social aspects	50.00 (50.00-75.00)	50.00 (37.50-62.50)	0.272b
Mental health	68.81±13.89	71.14±4.43	0.769^a^
Anthropometrics measurements			
BMI (kg/m²)	32.21±3.17	32.04±3.41	0.574^a^
NC (cm)	42.55±2.50	42.30±2.93	0.383^a^
WC (cm)	110.13±9.27	109.41±9.49	0.439^a^
HC (cm)	111.52±10.60	111.40±11.01	0.901^a^
WHR (cm)	0.99±0.04	0.97±0.02	0.176^a^

AHI: Apnea-hypopnea index; SpO_2_: Peripheral oxygen saturation; ODI: Oxygen desaturation index; BMI: Body mass index; NC: Neck circumference; WC: Waist circumference; HC: Hip circumference; WHR: Waist-to-hip ratio; Data expressed as mean and standard deviation and median and interquartile range.

a T test of paired samples;

b Wilcoxon; Significant values with *p*<0.05.

In addition, an association was made between AHI and the level of sleepiness obtained on the ESS and QoL (Table 3). It should be noted that there was a negative and moderate correlation between AHI and functional capacity (r=-0.477; *p*=0.029) and vitality (r=0.555; *p*=0.009) in the evaluated sample.

**Table 3:** Association between apnea-hypopnea index and level of sleepiness and quality of life.

	IAH	
	r	p
Epworth	0.304	0.169
Functional capacity	-0.477	0.029*
Physical limitations	0.063	0.785
Pain	0.253	0.268
General state	0.013	0.955
Vitality	-0.555	0.009*
Emotional aspects	0.259	0.258
Social aspects	0.306	0.178
Mental health	-0.010	0.966

AHI: Apnea-hypopnea index; Spearman's correlation (p<0.05).

## DISCUSSION

The present study showed a high prevalence of OSAS in the evaluated sample and that intercity transport drivers diagnosed with OSAS who underwent MAD showed a significant reduction in the apnea and hypopnea rates without any difference in sleepiness and quality of life having been evidenced, except in the pain domain. However, the evaluated individuals showed a perception of change after the use of MAD being reflected in their perceived improvement.

OSAS is associated with increased morbidity and mortality from cardiovascular disease and traffic accidents. The prevalence of OSAS currently varies from 9 to 38% in the adult population, with a higher occurrence in men (13 to 33%) than in women (6 to 19%)^19^. There was an OSAS prevalence of 63.90% among the evaluated public transport drivers in our study, and 86.36% of these were overweight and obese^19^. Studies point out that aging, male gender, increased neck circumference, and excess weight increase the risk of developing OSAS^6^^,^^19^^-^^21^. The higher prevalence of OSAS in males occurs due to the central fat distribution, whereas peripheral adiposity and the absence of testosterone protect women from this occurrence^22^. A high prevalence of OSAS stands out^23^ in relation to public transport drivers. According to Alahmaria et al.^23^, most drivers accidentally fall asleep at least once while driving and that poor quality sleep is considered a predictor of traffic accidents.

There is strong evidence for indicating intraoral devices in patients who snore and are unsuccessful in conservative treatment such as weight loss, avoiding alcohol, positional therapy), as well as in cases where there is a recommendation to prescribe intraoral devices. The same study points to a moderate degree of evidence or use of intraoral devices for patients who are intolerant to the use of CPAP and further comparing the use of personalized x non-personalized intraoral devices found in a weak form of the disease, but which can be used using custom appliances^24^.

In comparing the use of CPAP and prefabricated MAD in patients with OSAS and of similar age to our study, Banhiran et al.^25^ showed that both presented a reduction in AHI and QoL after 6 weeks of intervention; however, it is inferred that CPAP is more effective in reducing respiratory parameters. In addition, Makihara et al.^26^ observed that 90.9% of patients who used MAD had improvements in AHI, peripheral oxygen saturation, and in subjective symptoms such as snoring, daytime sleepiness, difficulty waking up, duration of apnea, and morning headache^26^. However, the same study emphasizes that when the patient does not obtain favorable results in the use of non- personalized MAD, the use of personalized devices is recommended as they favor better adjustments to the patient^26^.

A study by Gagnadoux et al.^18^ showed a significant reduction in AHI and ESS after 6 months of treatment in patients with mild to severe OSAS, in which they also point out that there was no significant difference in the treatment of thermoplastic and personalized MAD. In addition, these MAD models become cheap treatments for patients with OSAS or even for those who fail positive therapy^27^. Therefore, the side effects of using non-personalized MAD are greater, such as discomfort and pain in the teeth and jaw, excessive salivation, pain in the teeth and pain in the oral tissue region and self- reported occlusal changes^18^^,^^25^^,^^26^, which corroborates with the worsening of the SF-36 pain domain after using the device in our study.

In view of the main complaints such as snoring, tiredness and daytime sleepiness reported by patients with OSAS, an adequate assessment of sleepiness becomes necessary, and the ESS is a reliable and valid instrument^12^. Commercial vehicle drivers have a higher prevalence of developing OSAS and insomnia compared to the general population, and daytime sleepiness is associated with increased BMI, depression and short sleep duration^28^. Ibrahimi and Laabouri^17^ evaluated the changes in AHI and daytime sleepiness after treatment with MAD and showed that this resource constitutes an effective treatment in OSAS, suggesting a reduction in sleepiness after the use of MAD. Bahammam et al.^7^ determined the prevalence of accidents related to drowsiness in male drivers over 18 years old and observed a score of 7.2 points on the ESS in the total sample, with 19.4% having a score ≥10 points, reinforcing that drowsiness should be considered as a warning sign and a risk factor for the occurrence of automobile accidents. Moreover, Viegas and Oliveira^29^ found that 27.5% of patients with daytime sleepiness had a score ≥10 points on the ESS.

Difficulty in their personal relationship with their partner, interrupted sleep, poor sleep quality, snoring, depression, reduced work productivity, excessive sleepiness during the day, a feeling of unrestful sleep and even increased risks for traffic accidents directly impact the QoL of individuals with OSAS. Thus, treatment with MAD improves health conditions and leads to increased willingness and energy to develop daily tasks, increased productivity, improved mood, reduced nighttime awakenings, morning headaches, blood pressure, and the occurrence cardiovascular diseases, all resulting in a better QoL^8^^,^^30^^,^^31^. Therapies such as the adoption of personalized and treaTable MAD reduce the intensity and frequency of snoring, promote an improvement in the sleep quality of individuals with OSAS and also of their partners, and should be used in those who are unsuccessful in weight loss measures, positional therapy and difficulty in avoiding alcohol consumption^24^.

There are limitations in the present study which should be highlighted, such as the fact that the study design does not enable comparison with a control group and the fact that the intraoral device is not fully customized, so even though the implemented model allows individualization when heating the device and molding it to their teeth, this capacity presents the limit imposed by the rigid external structural part. In addition to the above, the reduced adhesion of the selected drivers made it difficult to obtain a larger sample size. However, it is noteworthy that our study identified OSAS occurrence in more than half of the intercity public transport drivers evaluated, and it was found that a pre-fabricated mandibular advancement device reduced the apnea-hypopnea index, without impacting a change in QoL and drowsiness.

## References

[1] Dempsey JA, Veasey SC, Morgan BJ, O'Donnell CP (2010). Pathophysiology of sleep apnea. Physiol Rev.

[2] Mediano O, Romero-Peralta S, Resano P, Cano-Pumarega I, Sánchez-de-la-Torre M, Castillo-García M (2019). Obstructive sleep apnea: emerging treatments targeting the
genioglossus muscle. J Clin Med.

[3] Nogueira F, Nigro C, Cambursano H, Borsini E, Silio J, Ávila J (2013). Guías prácticas de diagnóstico y tratamiento
del síndrome de apneas e hipopneas obstructivas del
sueño. Medicina.

[4] Watson NF, Badr MS, Belenky G, Bliwise DL, Buxton OM, Buysse D (2015). Recommended amount of sleep for a healthy adult: a joint
consensus statement of the American academy of sleep medicine and sleep
research society. J Clinic Sleep Med.

[5] Fonseca MIP, Pereira T, Caseiro P (2015). Mortalidade e incapacidade em pacientes com apneia do sono: uma
metanálise. Arq Bras Cardiol.

[6] Guimarães C, Martins MV, Rodrigues LV, Teixeira F, Santos JM (2012). Escala de sonolência de Epworth na síndrome de
apneia obstrutiva do sono: uma subjetividade subestimada. Rev Port Pneumol.

[7] BaHammam AS, Alkhunizam MA, Lesloum RH, Alshanqiti AM, Aldakhil AM, Pandi-Perumal SR (2014). Prevalence of sleep-related accidents among drivers in Saudi
Arabia. Ann Thorac Med..

[8] Campostrini DDA, Prado LBF, Prado GF (2014). Síndrome da apneia obstrutiva do sono e doenças
cardiovasculares. Rev Neurociênc.

[9] Haddad FLM, Vidigal TA, Mello-Fujita L, Cintra FD, Gregório LC, Tufik S (2013). The influence of nasal abnormalities in adherence to continuous
positive airway pressure device therapy in obstructive sleep apnea
patients. Sleep Breath.

[10] Kushida CA, Morgenthaler TL, Littner MR, Alessi CA, Bailey D, Coleman JRJ (2006). Practice parameters for the treatment of snoring and obstructive
sleep Apnea with oral appliances: an update for 2005. Sleep.

[11] Aarab G, Lobbezoo F, Hamburger HL, Naeije M (2011). Oral appliance therapy versus nasal continuous positive airway
pressure in obstructive sleep apnea: a randomized, placebo-controlled
trial. Respiration.

[12] Bertolazi AN, Fagondes SC, Hoff LS, Pedro VD, Barreto SSM, Johns MW (2009). Validação da escala de sonolência de Epworth
em português para uso no Brasil. J Bras Pneumol.

[13] Ciconelli RM, Ferraz MB, Santos W, Meinão L, Quaresma MR (1999). Tradução para a língua portuguesa e
validação do questionário genérico de
avaliação de qualidade de vida SF-36 (Brasil SF-36).
Brazilian-Portuguese version of the SF-36. A reliable and valid quality of
life outcome measure. Rev Bras Reumatol.

[14] Zhao YY, Wang R, Gleason KJ, Lewis EF, Quan SF, Toth CM (2017). Effect of continuous positive airway pressure treatment on
health-related quality of life and sleepiness in high cardiovascular risk
individuals with sleep apnea: best apnea interventions for research
(BestAIR) trial. Sleep.

[15] Domingues L, Cruz E (2012). Adaptação cultural e contributo para a
validação da Escala Patient Global Impression of
Change. IfisiOnline.

[16] Zancanella E, Haddad FM, Oliveira LAMP, Nakasato A, Duarte BB, Soares CFP (2012). Apneia obstrutiva do sono e ronco primário:
diagnóstico.

[17] Ibrahimi ME, Laabouri M (2016). Pilot study of a new adjusTable thermoplastic mandibular
advancement device for the management of obstructive sleep apnoea- hypopnoea
syndrome: a brief research letter. Open Respir Med J.

[18] Gagnadoux F, Nguyen XL, Le Vaillant M, Priou P, Meslier N, Eberlein A (2017). Comparison of titrable thermoplastic versus custom-made
mandibular advancement device for the treatment of obstructive sleep
apnoea. Respir Med.

[19] Senaratna CV, Perret JL, Lodge C, Adrian L, Campbell BE, Matheson MC (2017). Prevalence of obstructive sleep apnea in the general population:
a systematic review. Sleep Med Rev.

[20] Mirrakhimov AE, Sooronbaev T, Mirrakhimov EM (2013). Prevalence of obstructive sleep apnea in Asian adults: a
systematic review of the literature. BMC Pulmonary Med.

[21] Arnardottir ES, Bjornsdottir E, Olafsdottir KA, Benediktsdottir B, Gislason T (2016). Obstructive sleep apnoea in the general population: highly
prevalent but minimal symptoms. Eur Respir J.

[22] Burgos RA, Carvalho GA (2012). Síndrome da apneia obstrutiva do sono (SAOS) e
sonolência diurna excessiva (SDE): influência sobre os riscos
e eventos de queda em idosos. Fisioter Mov..

[23] Alahmaria MDS, Alanazib TM, Batawib AA, Al-Osaimib EA, Alrabeeaha S, Jebakumara Z (2019). Sleepy driving and risk of obstructive sleep apnea among truck
drivers in Saudi Arabia. Traffic Inj Prev.

[24] Ramar K, Dort LC, Katz SG, Lettieri CJ, Harrod CG, Thomas SM (2015). Clinical practice guideline for the treatment of obstructive
sleep apnea and snoring with oral appliance therapy: an update for 2015: an
American Academy of Sleep Medicine and American Academy of Dental Sleep
Medicine Clinical Practice Guideline. J Clin Sleep Med..

[25] Banhiran W, Assanasen P, Nopmaneejumrudlers C, Nujchanart N, Srechareon W, Chongkolwatana C (2018). AdjusTable thermoplastic oral appliance versus positive airway
pressure for obstructive sleep apnea. Laryngoscope.

[26] Makihara E, Kawano T, Miyajima R, Masumi S, Enciso R, Clark G (2016). Assessment of oral appliance for obstructive sleep apnea
patients. Clin Exp Dent Res.

[27] Friedman M, Pulver T, Wilson MN, Golbin D, Leesman C, Lee G (2010). Otolaryngology office-based treatment of obstructive sleep
apnea-hypopnea syndrome with titraTable and non titraTable thermoplastic
mandibular advancement devices. Otolaryngol Head Neck Surg.

[28] Sunwoo JS, Shin DS, Hwangbo Y, Kim WJ, Chu MK, Yun CH (2019). High risk of obstructive sleep apnea, insomnia, and daytime
sleepiness among commercial motor vehicle drivers. Sleep Breath.

[29] Viegas CAA, Oliveira HW (2006). Prevalência de fatores de risco para a síndrome da
apneia obstrutiva do sono em motoristas de ônibus
interestadual. J Bras Pneumol..

[30] Dal-Fabbro C, Bittencourt LRA, Chaves CM, Dal-Fabbro C, Chaves CM, Tufik S (2011). Classificação dos distúrbios do
sono. A odontologia na medicina do sono.

[31] Morsy NE, Farrag NS, Zaki NFW, Badawy AY, Abdelhafez SA, El-Gilany AH (2019). Obstructive sleep apnea: personal, societal, public health, and
legal implications. Rev Environ Health.

